# The genetic architecture of complete blood counts in lactating Holstein dairy cows

**DOI:** 10.3389/fgene.2024.1360295

**Published:** 2024-03-27

**Authors:** Cori J. Siberski-Cooper, Mary S. Mayes, Patrick J. Gorden, Luke Kramer, Vishesh Bhatia, James E. Koltes

**Affiliations:** ^1^ Department of Animal Science, Iowa State University, Ames, IA, United States; ^2^ Veterinary Diagnostic and Production Animal Medicine, Iowa State University, Ames, IA, United States

**Keywords:** dairy cattle, complete blood counts, genome-wide association study, candidate genes, heritability

## Abstract

Complete blood counts (CBCs) measure the abundance of individual immune cells, red blood cells, and related measures such as platelets in circulating blood. These measures can indicate the health status of an animal; thus, baseline circulating levels in a healthy animal may be related to the productive life, resilience, and production efficiency of cattle. The objective of this study is to determine the heritability of CBC traits and identify genomic regions that are associated with CBC measurements in lactating Holstein dairy cattle. The heritability of CBCs was estimated using a Bayes C0 model. The study population consisted of 388 cows with genotypes at roughly 75,000 markers and 16 different CBC phenotypes taken at one to three time points (n = 33, 131, and 224 for 1, 2, and 3 time points, respectively). Heritabilities ranged from 0.00 ± 0.00 (red cell distribution width) to 0.68 ± 0.06 (lymphocytes). A total of 96 different 1-Mb windows were identified that explained more than 1% of the genetic variance for at least one CBC trait, with 10 windows explaining more than 1% of the genetic variance for two or more traits. Multiple genes in the identified regions have functions related to immune response, cell differentiation, anemia, and disease. Positional candidate genes include RAD52 motif-containing protein 1 (*RDM1*), which is correlated with the degree of immune infiltration of immune cells, and C-X-C motif chemokine ligand 12 (*CXCL12*), which is critically involved in neutrophil bone marrow storage and release regulation and enhances neutrophil migration. Since animal health directly impacts feed intake, understanding the genetics of CBCs may be useful in identifying more disease-resilient and feed-efficient dairy cattle. Identification of genes responsible for variation in CBCs will also help identify the variability in how dairy cattle defend against illness and injury.

## 1 Introduction

The health of dairy cattle is important for their welfare, efficiency, and profitability. Previous research has shown that health disorders impact feed intake, feed efficiency, and milk quality (Brown and Bradford, 2021; Lochmiller and Deerenberg, 2000; Siberski-Cooper et al., 2023). Given the relationships between health and feed intake and related traits, information on health could be useful to improve predictions of feed efficiency in dairy cattle. Currently, genetic evaluations for health traits have primarily used producer-reported data. This information can be highly subjective, and the recording consistency differs greatly from farm to farm, resulting in lower heritability estimates (König and May 2019). In addition to these complications, the way in which resilience is defined is not consistent. Therefore, implementation of selection for improved performance in response to illness in dairy cattle has not yet been feasible (Berghof et al., 2019). Identification of proxy traits that are objective and easy to measure would be beneficial for identification of animals that are more resilient and thus genetic selection for improved resilience and maintained efficiency under disease or stress. Complete blood counts (CBCs; Table 1) are good candidate indicator traits of health status due to their relationships with the immune system. Although no assay is perfect for general disease detection and surveillance, CBCs are routinely used for early diagnosis of illness in dairy cattle, making red and white blood cell counts from these assays routinely available as disease indicators (Roland et al., 2014). A limited number of studies have reported the relationship of CBCs with growth, disease, and efficiency in beef cattle and swine (Leach et al., 2013; Mpetile et al., 2015; Chinchilla-Vargas et al., 2020). Additionally, there is only one report on genomic heritabilities of CBCs in dairy cattle (Siberski-Cooper et al., 2022) and one known study reporting non-genomic heritability estimates of specific blood leukocyte types in Holstein Friesian cattle (Denholm et al., 2017). Thus, the objectives of this study are to 1) estimate the genomic heritability of CBCs in lactating Holstein cows and 2) identify genomic regions associated with CBCs.

**TABLE 1:** Measurements reported in complete blood counts.

Measure	Abbreviation	Unit	Transformation
White blood cells	WBC	K/uL	Log
Neutrophils	NEUT	K/uL	Log
Lymphocytes	LYMPH	K/uL	Log
Monocytes	MONO	K/uL	—
Eosinophils	EOSI	K/uL	Log
Basophils	BASO	K/uL	Log
Large unstained cells	LUC	K/uL	Log
Red blood cells	RBC	M/uL	—
Hemoglobin	HGB	g/dL	—
Hematocrit	HCT	%	—
Mean corpuscular volume	MCV	fL	Log
Mean corpuscular hemoglobin	MCH	Pg	Log
MCH concentration	MCHC	g/dL	—
Red cell distribution width	RDW	%	Box Cox
Platelets	PLT	K/uL	—
Mean platelet volume	MPV	fL	Box Cox

## 2 Materials and methods

### 2.1 Animal husbandry

All research conducted in this study was approved by the Iowa State University Animal Care and Use Committee (IACUC) under protocols 18–174 and 21–144. Data of 418 Holstein cows were collected between 2020 and 2022. Cows ranged from first through sixth parity and were 24–272 days in milk (DIM) at the start of data collection. To classify the point in lactation of the cows, DIM were assigned to one of four groups, namely, early-, peak-, mid-, and late-lactation. Early lactation was defined as DIM prior to 50 days, peak lactation was considered DIM 51–90 days, DIM 91–200 days were classified as mid-lactation, and any DIM after 201 days were assigned to the late-lactation category. Cows were grouped into contemporary groups (CGs) based on barn location (i.e., pen) and study replicates based on dates of data collection, resulting in a total of 10 CGs in the study. Cows were housed in a free stall barn at the Iowa State University (ISU) Dairy Farm. All cows received a standard total mixed ration (TMR) diet containing corn silage, alfalfa hay, whole cottonseed, molasses, ground corn, soybean meal and hulls, dried distiller grains, and a mineral and protein mix. Cows were milked twice per day, and milk samples were collected at both milkings 1 day per week. Milk samples were used in conjunction with daily milk weight to approximate the daily fat and protein yields.

### 2.2 Phenotype collection

Blood samples were collected for CBC measurements from cows two or three times during the trial period, with the number of blood collections depending on the CG. Blood samples were taken in the morning, prior to feeding and milking. Sampling occurred at the start and end of each trial, with CGs 1 to 8 having an additional sample taken at roughly halfway through the collection period. The average time between samples was 21 days. A total of 33 cows had CBCs at one time point, 131 at two time points, and 224 at three time points. Some cows had a single CBC due to either clotting of the blood sample, which prohibits the analysis for CBCs, or removal from the study pen due to a severe health disorder, including clinical mastitis and respiratory disease, based on daily consultation with ISU veterinary staff. Four milliliters of blood was collected from the tails of cattle and added to an EDTA blood tube and analyzed for CBCs using an ADVIA^®^ 2120 Hematology System (Siemens Healthineers, Erlangen, Germany) as a commercial diagnostic service at the ISU Veterinary Clinical Pathology Laboratory. Of the 418 cows from which data were collected, CBCs were successfully reported for a total of 414 cows. A full list of cell abundance phenotypes from CBCs, along with the cells’ full names and abbreviations, is provided in Table 1. The distribution of each CBC phenotype was assessed for normality using a quantile–quantile plot. The WBC, MCV, MCH, BASO, EOSI, LYMPH, NEUT, and LUC were log-transformed to better approximate a normal distribution, whereas RDW and MPV required a Box–Cox transformation (lambda = −2). All other traits were not transformed. A summary of the cell count measures, including the mean and standard deviation, can be found in Supplementary Table S1.

### 2.3 Genotypic information

Genotypic data consisted of 78,985 SNP markers across the genome. Marker positions were obtained for the ARS-UCD 1.2 bovine genome build. Genotypic data quality control required a SNP marker call rate greater than 95% and a minor allele frequency greater than 5%. A total of 388 animals and 75,823 SNPs remained in the dataset following quality control.

### 2.4 Genetic analyses

#### 2.4.1 Estimates of narrow sense heritability and repeatability from genomic data

Following the methodology presented in Bhatia et al. (2023), estimates of heritability for traits were obtained using the following univariate trait-based Bayes-C0 model (Kizilkaya et al., 2010):

$$y_{ijkl}={CG}_{i}+{DIMwindow}_{j}+{Par}_{k}+PE\left({Cow}_{l}\right)+\sum_{n=1}^{p}m_{ijkln}\beta_{n}+e_{ijkl},$$

where $y_{ijkl}$ is the CBC phenotype of cow ijkl ; ${CG}_{i}$ is the class effect of the contemporary group effect ( i=1,…,10 ); ${DIMwindow}_{j}$ is the class effect of the point in lactation (early, peak, mid, or late); ${Par}_{k}$ is the class effect of lactation number ( k = 1, 2, or 3+); $PE\left({Cow}_{l}\right)$ is the random permanent environmental effect of the cow to account for repeated records; $PE\left({Cow}_{l}\right)\;∼\;N\left(0,{\;\sigma }_{PE}^{2}\right)$ , where ${\;\sigma }_{PE}^{2}$ is the permanent environmental variance; $m_{ijkln}$ is the genotype for SNP n (coded as 0, 1, or 2) with a total of p SNPs, with allele substitution effect $\beta_{n}$ . These analyses were completed using the Julia for Whole-genome Analysis Software (JWAS; Cheng et al., 2018) using a Markov Chain Monte Carlo (MCMC) method of 80,000 iterations for all CBC traits except PLT, which required a chain length of 150,000 iterations to reach convergence, based on a trace plot. A burn-in of 5,000 samples was used for all analyses. For each trait, the sampled genome-wide genetic, permanent environmental and residual variances were saved for every 100th iteration and used to compute the samples of the posterior distribution of the repeatability. This was calculated by dividing the sum of the sampled genetic and permanent environmental variances by the sampled phenotypic variance (i.e., the sum of the sampled genetic, permanent environmental and residual variances). Using the ‘coda’ package in R (Plummer et al., 2015), the mean and 95% highest posterior density (HPD) of the repeatability were obtained.

#### 2.4.2 Genome-wide association study

Following Bhatia et al. (2023), a univariate marker-based Bayes-B model was implemented in JWAS to identify genomic regions associated with CBC measurements. The model used was

$$y_{ijkl}={CG}_{i}+{DIMwindow}_{j}+{Par}_{k}+PE\left({Cow}_{l}\right)\;\sum_{n=1}^{p}m_{ijkln}\beta_{n}\delta_{n}+e_{ijkl},$$

where parameters follow the same naming convention described above, with the addition of $\delta_{n}$ . $\delta_{n}$ indicates whether SNP n was included within the model ( $\delta_{n}=1$ ) or not ( $\delta_{n}=0$ ) for an iteration of the model. The prior probability of exclusion (π) and prior variances (presented in Table 2) were estimated using a Bayes Cπ model (Kizilkaya et al., 2010). An MCMC length and burn-in were set as described for heritability estimates. From every 100th iteration, sample breeding values and 1-Mb non-overlapping windows based on the reference genome ARS-UCD 1.2 were used to obtain the samples of the posterior distributions of genome-wide and window-based genetic variance. These samples were used to estimate the percent of genetic variance explained and the window-based posterior probabilities of association (WPPA; Fernando et al., 2017), which provide information about the level of certainty that the window was included in the posterior distribution, for each 1-Mb window.

**TABLE 2:** Prior probability of exclusion (π) and variance estimates of complete blood count measures estimated from Bayes Cπ.

Measure	π	Genetic variance	Residual variance
White blood cells^a^	0.998	0.02	0.02
Neutrophils^a^	0.999	0.02	0.06
Lymphocytes^a^	0.998	0.05	0.01
Monocytes	0.998	3.17e-03	0.01
Eosinophils^a^	0.999	0.14	0.26
Basophils^a^	0.999	0.07	0.05
Large unstained cells^a^	0.998	0.03	0.25
Red blood cells	0.998	0.13	0.07
Hemoglobin	0.998	0.21	0.19
Hematocrit	0.998	1.45	1.37
Mean corpuscular volume^a^	0.998	2.89e-03	2.94e-04
Mean corpuscular hemoglobin^a^	0.998	2.56e-03	3.25e-04
Mean corpuscular hemoglobin concentration	0.999	0.08	0.28
Red cell distribution width^b^	0.998	1.01e-08	6.16e-09
Platelets	0.999	2574.96	10559.44
Mean platelet volume^b^	0.999	1.99e-06	2.20e-06

^a^ Log-transformed.

^b^ Box–Cox-transformed.

#### 2.4.3 Identification of overlapping quantitative trait loci and candidate genes for CBC traits

Quantitative trait loci (QTLs) were identified as 1-Mb windows of the genome explaining more than 1% of the genetic variation, which contained all genotyped SNPs within the window. In order to explore whether the identified QTL explaining the most variance for each CBC trait had previously been associated with disease and other production traits in cattle, the GWAS peak (i.e., the 1-Mb region) was identified, and the 3′ and 5′ flanking 1-Mb windows were examined in the Animal Quantitative Trait Locus Database (QTLdb; Hu et al., 2022), based on the genomic coordinates defined at the QTLdb. The region explaining the most variance for lymphocytes was unable to be explored due to the inability to view chromosomal information on the QTLdb.

Potential candidate genes were identified by examining the 1-Mb region in the Ensembl genome browser. Previous findings related to the genes contained in the window were researched using NCBI, GeneCards, and previous publications (Stelzer et al., 2016; Cunningham et al., 2022; Sayers et al., 2022). If there were few or no genes in the 1-Mb window associated with the trait, the flanking 1-Mb windows were also inspected. Due to the number of QTLs identified, the 1-Mb region explaining the most genetic variance was explored for each CBC trait.

## 3 Results

### 3.1 Heritability and repeatability estimates

Genome-based heritability estimates for all CBC traits are reported in Table 3. Heritability estimates ranged from 0.00 ± 0.00 (RDW) to 0.68 ± 0.06 (LYMPH). The majority of the CBC traits had moderate heritability estimates, with a few having heritability estimates that are considerably high (i.e., larger than 0.50; RBC and LYMPH). The mean repeatability and upper and lower bounds of the HPD of repeatability for each trait are presented in Table 4. Repeatability estimates for CBC traits were typically moderate to high, with some having low repeatability. The range in mean repeatability was 0.00 (RDW) to 0.84 (LYMPH). Traits that had higher heritability tended to also have higher repeatability.

**TABLE 3:** Heritability estimates of complete blood count measures.

Measure	Heritability ± standard error
White blood cells^a^	0.44 ± 0.07
Neutrophils^a^	0.20 ± 0.04
Lymphocytes^a^	0.68 ± 0.06
Monocytes	0.19 ± 0.05
Eosinophils^a^	0.32 ± 0.05
Basophils^a^	0.48 ± 0.05
Large unstained cells^a^	0.09 ± 0.03
Red blood cells	0.56 ± 0.04
Hemoglobin	0.39 ± 0.06
Hematocrit	0.41 ± 0.05
Mean corpuscular volume^a^	0.20 ± 0.03
Mean corpuscular hemoglobin^a^	0.26 ± 0.04
MCH concentration	0.05 ± 0.02
Red cell distribution width^b^	0.00 ± 0.00
Platelets	0.18 ± 0.04
Mean platelet volume^b^	0.02 ± 0.01

^a^ Log-transformed.

^b^ Box–Cox-transformed.

**TABLE 4:** Mean repeatability estimates and 95% confidence interval of repeatability for complete blood count measures.

Measure	Mean repeatability	95% confidence interval
White blood cells^a^	0.60	0.54–0.65
Neutrophils^a^	0.33	0.25–0.39
Lymphocytes^a^	0.84	0.81–0.86
Monocytes	0.31	0.25–0.38
Eosinophils^a^	0.42	0.35–0.48
Basophils^a^	0.60	0.56–0.65
Large unstained cells^a^	0.31	0.25–0.38
Red blood cells	0.63	0.59–0.68
Hemoglobin	0.51	0.45–0.57
Hematocrit	0.50	0.44–0.56
Mean corpuscular volume^a^	0.23	0.17–0.29
Mean corpuscular hemoglobin^a^	0.30	0.23–0.37
MCH concentration	0.09	0.05–0.13
Red cell distribution width^b^	0.00	0.00–0.00
Platelets	0.28	0.21–0.34
Mean platelet volume^b^	0.03	0.01–0.05

^a^ Log-transformed.

^b^ Box–Cox-transformed.

### 3.2 Genomic regions associated and overlapping QTLs with CBC traits

**FIGURE 1:**
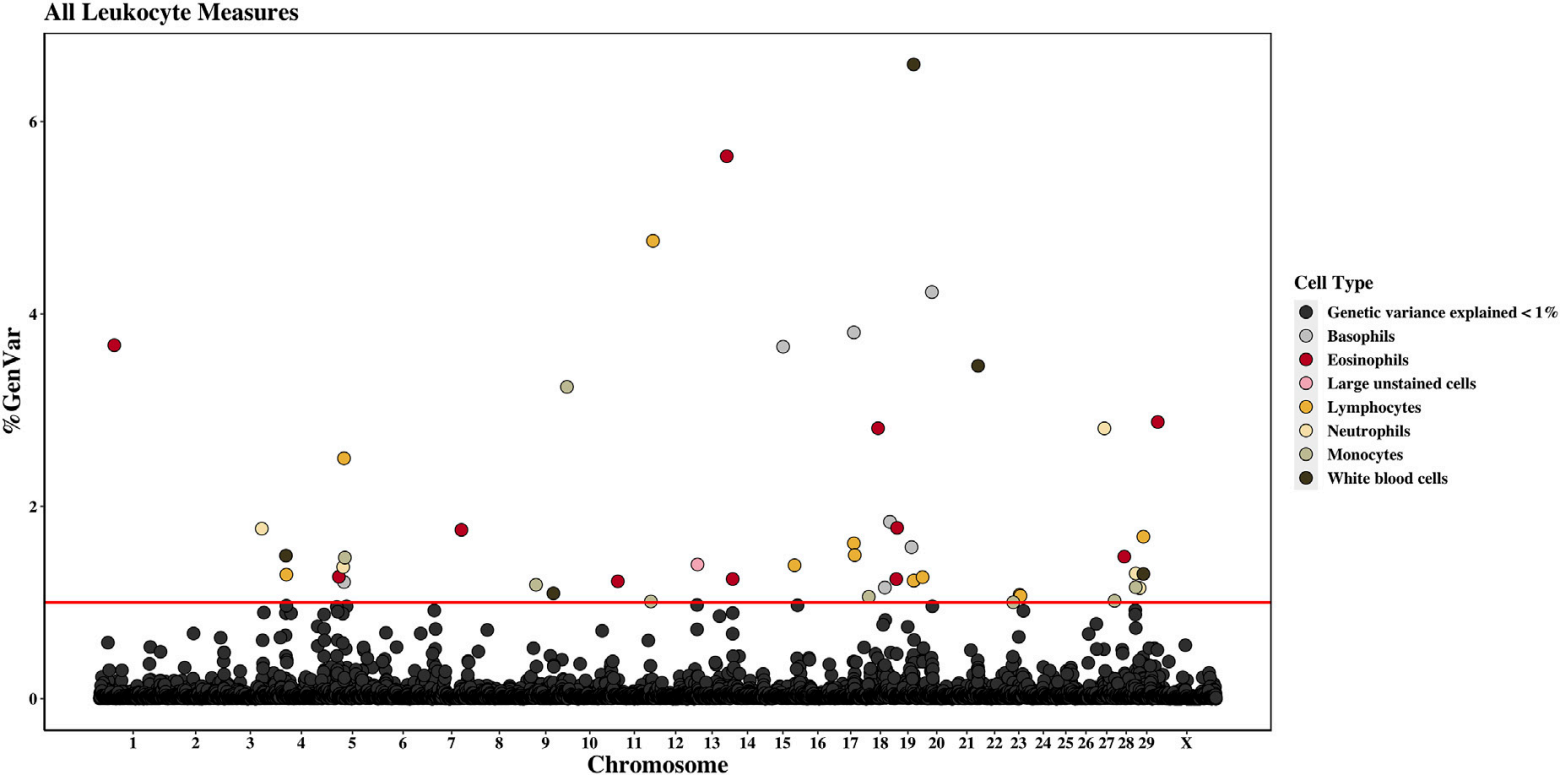
Genome-wide association study results for all white blood cells and white blood cell sub-types (i.e., leukocyte traits), with chromosome number on the *X*-axis and the percent of genetic variance explained on the *Y*-axis. Each point represents a 1-Mb window, and the red line is at 1% of the genetic variance explained.

**FIGURE 2:**
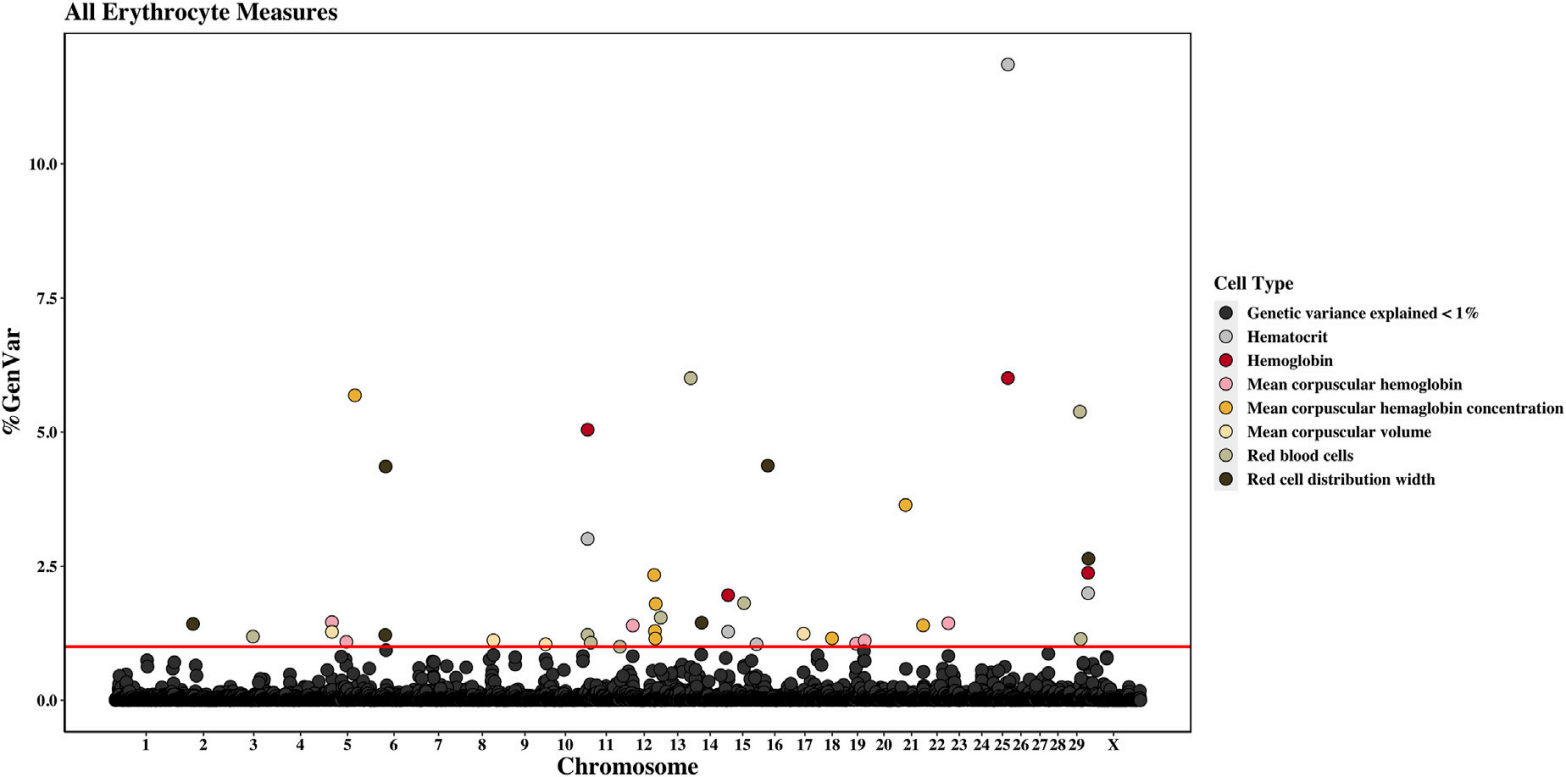
Genome-wide association study results for all red blood cells and red blood cell-related measures (i.e., erythrocyte traits), with chromosome number on the *X*-axis and the percent of genetic variance explained on the *Y*-axis. Each point represents a 1-Mb window, and the red line is at 1% of the genetic variance explained.

**FIGURE 3:**
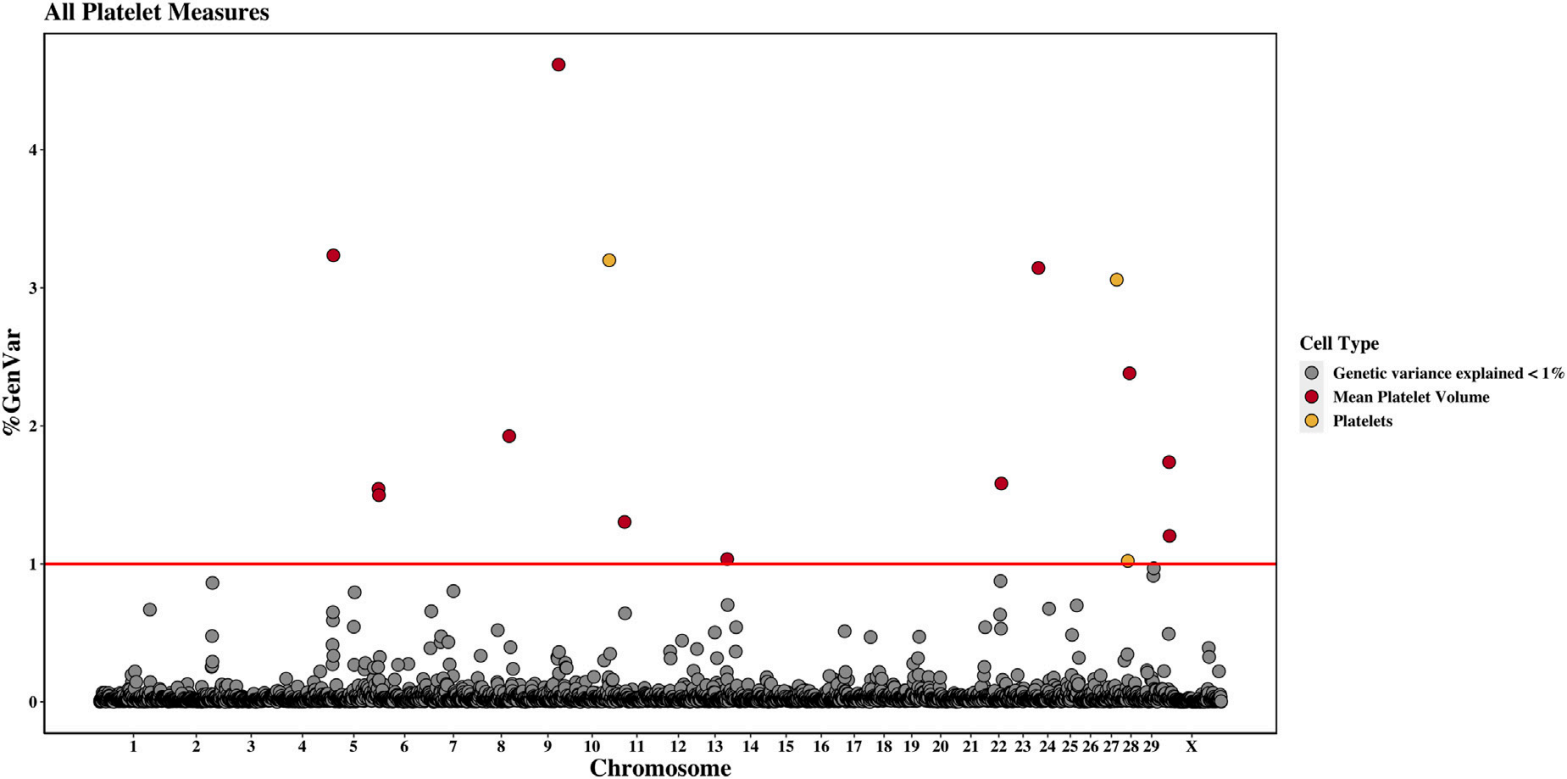
Genome-wide association study results for platelets and mean platelet volume, with chromosome number on the *X*-axis and the percent of genetic variance explained on the *Y*-axis. Each point represents a 1-Mb window, and the red line is at 1% of the genetic variance explained.

Across the genome, 95 unique 1-Mb windows explained more than 1% of the genetic variance for at least one CBC trait. There were nine 1-Mb windows that accounted for more than 1% of the genetic variance for two CBC traits, and one 1-Mb window was found for three CBC traits. Each chromosome had at least one 1-Mb significant window for at least one trait, except for chromosome 26. Table 5 presents the locations of the windows that explained the most genetic variance, the percentage of genetic variance explained by the window, the WPPA, and potential candidate genes within the window for each chromosome. For a list of all 1-Mb windows that explained more than 1% of the genetic variance of the CBC traits, see Supplementary Table S2. Manhattan plots of the results of the GWAS for white blood cell, red blood cell, and platelet traits are shown in Figure 1, Figure 2, and Figure 3, respectively. The most genetic variance explained (11.9%) was by the window located at Mb 35 on chromosome 25 and was associated with hematocrit percentage. The average WPPA was 0.38, and a total of 22 windows had a WPPA above 0.50.

**TABLE 5:** Summary of 1-Mb windows explaining the most genetic variance for each complete blood count trait.

Trait	Chromosome	Mb^a^ window	Number of SNPs in the window	WPPA^b^	% genetic variance explained	Candidate gene(s)
White blood cells^c^	19	45–46	44	0.70	6.6	*RDM1*, *KANSL1*, and *CDC27*
Neutrophils^c^	27	17–18	37	0.31	2.8	*CXCL12*
Lymphocytes^c^	11	97–98	37	0.75	4.8	*ZBTB34* and *ZBTB43*
Monocytes	9	102–103	65	0.40	3.2	*SMOC2*
Eosinophils^c^	13	76–77	42	0.63	5.6	*SULF2*
Basophils^c^	20	24–25	28	0.65	4.2	*GZMA* and *GZMK*
Large unstained cells^c^	13	7–8	27	0.21	1.4	*TASP1*
Red blood cells	13	75–76	44	0.94	6.0	*EYA2*, *OCSTAMP*, and *TP53RK*
Hemoglobin	25	35–36	45	0.61	6.0	*EPO*, *AChE*, and *TFR2*
Hematocrit	25	35–36	45	0.82	11.9	*EPO*, *AChE*, and *TFR2*
Mean corpuscular volume^c^	5	20–21	26	0.58	1.3	–
Mean corpuscular hemoglobin^c^	5	20–21	26	0.60	1.5	–
Mean corpuscular hemoglobin concentration	5	79–80	25	0.54	5.7	–
Red cell distribution width^d^	16	22–23	25	0.36	4.4	*ABCG2* and *HERC6*
Platelets	10	92–93	39	0.28	3.2	*CEP128*
Mean platelet volume^d^	9	77–78	32	0.61	4.6	*HECA* and *CITED2*

^a^ Megabase.

^b^ Window-based posterior probabilities of association.

^c^ Log-transformed.

^d^ Box–Cox-transformed.

Within the 1-Mb window explaining the most genetic variance or flanking 1-Mb windows of thirteen CBC traits (WBC, RBC, HGB, HCT, MCV, MCH, RDW, MPV, NEUT, MONO, EOSI, BASO, and LUC), genome-wide marker associations (i.e., GWAS-based QTL) have previously been identified for production-related traits, such as fat yield or percentage, protein yield or percentage, milk conjugated linoleic acid content, and milk yield. Additionally, QTLs within the regions examined for RBC, RDW, MPV, NEUT, MONO, EOSI, BASO, and LUC have previously been identified for the length of productive life. Finally, RBC, RDW, MPV, NEUT, EOSI, BASO, and LUC had QTLs for net merit (Hu et al., 2022). For all overlapping QTLs discussed in the upcoming sections, the information on QTLs was obtained from the animal QTLdb (Hu et al., 2022). The results for all overlapping QTLs are presented in Supplementary Table S3.

#### 3.2.1 White blood cell count

A total of five 1-Mb windows were found to explain at least 1% of the genetic variation for WBC. These windows were located on chromosomes 4, 9, 19, 21, and 29. The window on chromosome 19 explained the most variance at 6.6% and was located at 45 Mb. The WPPA for this window was 0.70. Within this window or just inside the 1-Mb window beginning at 46 Mb are the RAD52 motif-containing protein 1 (*RDM1*), KAT8 regulatory NSL complex subunit 1 (*KANSL1*), and cell division cycle 27 (*CDC27*) genes.

#### 3.2.2 Red blood cell count

Nine 1-Mb windows located across six chromosomes explained 1.2%–6.0% of the genetic variance of RBCs. The regions identified were located on chromosomes 3, 11, 12, 13, 15, and 29, with three windows located on chromosome 11 and two on chromosome 29. The window on chromosome 13 at 75 Mb explained the most variance (6.0%) and had a WPPA of 0.94. Genes located in this window include EYA transcriptional coactivator and phosphatase (*EYA2*), osteoclast stimulatory transmembrane protein (*OCSTAMP*), and TP53 regulating kinase (*TP53RK*). Previously, a QTL was identified in the region on chromosome 13 located in the 76th Mb for somatic cell score (SCS). Within this region, QTLs were also identified for metabolic body weight (MBW) and average daily gain (ADG). Finally, a QTL located in the 74th Mb on chromosome 13 was previously identified for body weight gain (BWG).

#### 3.2.3 Hemoglobin amount and hematocrit percentage

Four 1-Mb windows were identified for HGB on chromosomes 11, 15, 25, and X. The same regions were identified for HCT, as well as an additional window on chromosome 15. For both traits, the region located at 35 Mb on chromosome 25 accounted for the largest amount of genetic variance. The window explained 6.0% of the variance for HGB and 11.9% for HCT. The WPPA was lower for this region for HGB, with a WPPA of 0.61 compared to that of 0.82 for HCT. The erythropoietin (*EPO*) gene and the acetylcholinesterase (*AChE*) and transferrin receptor 2 (TFR2) genes are located within this window. Two QTLs for ADG were previously identified in the 35th Mb of chromosome 25.

#### 3.2.4 Mean corpuscular volume

Between 1.1% and 1.3% of the genetic variance of MCV was accounted for by four 1-Mb windows located on chromosomes 5, 8, 10, and 17. The window on chromosome 5, located at 20 Mb, accounted for the most variance (1.3%). This window had a WPPA of 0.58.

#### 3.2.5 Mean corpuscular hemoglobin amount

Six windows on chromosomes 5, 12, 19, and 22 explained 1.1%–1.5% of the genetic variance of MCH. Two 1-Mb windows were identified on both chromosomes 5 and 19. The window on chromosome 5 at 20 Mb accounted for the largest amount of genetic variance at 1.5% and had a WPPA of 0.60.

#### 3.2.6 Mean corpuscular hemoglobin concentration

A total of eight 1-Mb windows explained at least 1% of the genetic variance of MCHC. These windows were located on chromosomes 5, 12 (four windows), 18, and 21 (two windows). The largest amount of variance (5.7%) was explained by the window located at 79 Mb on chromosome 5 and had a WPPA of 0.54. Five QTLs previously identified for bovine tuberculosis susceptibility and M. paratuberculosis susceptibility were located in the region in the 78th Mb on chromosome 5.

#### 3.2.7 Red cell distribution width

Six windows explained more than 1% of the genetic variance of RDW. These windows were located on chromosomes 2, 6 (two windows), 14, 16, and X. The most variance was explained by the window on chromosome 16 (22 Mb), accounting for 4.4% of the genetic variance. This window had a WPPA of 0.36. Within the genomic region at 21 Mb on chromosome 16, QTLs were previously identified for ADG and BWG, whereas a QTL was found for feed conversion ratio in the 23rd Mb. Within a window explaining 1.2% of the genetic variation in RDW located at chromosome 6 (36 Mb) are the ATP-binding cassette subfamily G member 2 (*ABCG2*) and HECT and RLD domain-containing E3 ubiquitin protein ligase family member 6 (*HERC6*) genes.

#### 3.2.8 Platelet count

Three 1-Mb regions, located on chromosomes 10, 27, and 28, were found to explain more than 1% of the genetic variance for PLT. The window located at 91 Mb on chromosome 10 accounted for the most variance (3.2%), and the WPPA of this window was 0.28. The gene centrosomal protein 128 (*CEP128*) is located in this window.

#### 3.2.9 Mean platelet volume

There were twelve windows found on nine chromosomes that explained between 1.0% and 4.6% of the genetic variance for MPV. Windows were located on chromosomes 5 (three windows), 8, 9, 11, 13, 22, 24, 28, and X (two windows). The most variance explained (4.6%) was for the window on chromosome 9 at 77 Mb, which had a WPPA of 0.61. The Hdc homolog, cell cycle regulator (*HECA*) gene is located just outside of the 77-Mb window, at approximately 76.8 Mb, and the Cbp/p300 interacting transactivator with Clu/Asp rich carboxy-terminal domain 2 (*CITED2*) is located within the window at 77 Mb. A QTL was previously identified for MBW in the 78th Mb.

#### 3.2.10 Neutrophil count

Five windows were identified on chromosomes 3, 5, 27, 28, and 29, explaining at least 1% of the genetic variance for NEUT. The window located at 17 Mb on chromosome 27 explained the most variance at 2.8%. This window had a WPPA of 0.31. Two QTLs were previously identified for ADG, one located within the 17-Mb and the other in the 18-Mb region. Additionally, a QTL was found for BWG in the 17th Mb and for dry matter intake in the 18th Mb. Within the window located at 45 Mb on chromosome 28, explaining 1.3% of the genetic variation, is the C-X-C motif chemokine ligand 12 (*CXCL12*) gene.

#### 3.2.11 Lymphocyte count

There were eleven windows that explained at least 1% of the genetic variance in LYMPH. These windows were located on chromosomes 4, 5, 11, 15, 17, 19, 20, 23, and 29. There were two windows located on both chromosomes 17 and 23. The largest amount of variance explained was 4.8% by the window located on chromosome 11 at 97 Mb. This window had a WPPA of 0.75. Located in this window are the zinc finger and BTB domain-containing 34 and 43 (*ZBTB34* and *ZBTB43*) genes.

#### 3.2.12 Monocyte count

Eight windows explained more than 1% of the genetic variance of MONO. Windows were found on chromosomes 5, 9 (two windows), 11, 18, 23, 27, and 28. The most variance was explained by the window on chromosome 9 beginning at 102 Mb (3.2%) and having a PPA of 0.40. The SPARC-related modular calcium-binding 2 (*SMOC2*) gene is found within this window. A QTL was previously identified in this region (chromosome 9, 102 Mb) for bovine respiratory disease (BRD) susceptibility. Moreover, within the 103-Mb region, a QTL was found for BWG.

#### 3.2.13 Eosinophil count

Between 1.2% and 5.6% of the genetic variance was explained by eleven windows for EOSI. Windows were located on chromosomes 1, 5, 7, 11, 13, 14, 18, 19, 28, and X, with two regions located on chromosome 19. The largest portion of variance (5.6%) explained was by the window located on chromosome 13 at 76 Mb. The WPPA of this window was 0.63. The sulfatase 2 (*SULF2*) gene is found within this window. Previously, QTLs were identified for SCS and clinical mastitis in the region located at 77 Mb on chromosome 13. Additionally, a QTL for BWG was found in the region at 76 Mb.

#### 3.2.14 Basophil count

Eight windows located on chromosomes 5, 15, 17, 18 (two windows), 19 (two windows), and 20 accounted for more than 1% of the genetic variance for BASO. The most variance explained was by the window on chromosome 20 (24th Mb), accounting for 4.2%. This window had a WPPA of 0.65. The granzyme A (*GZMA*) and K (*GZMK*) genes are found in this window. A QTL was previously identified for clinical mastitis in the 23rd Mb and for SCS in the 25th Mb on chromosome 20.

#### 3.2.15 Large unstained cells count

A single 1-Mb window located on chromosome 13 beginning at the seventh Mb accounted for 1.4% of the genetic variance in large unstained cells. The WPPA of this window was 0.20. The taspase 1 (*TASP1*) gene was found in this window.

## 4 Discussion

This is the first study describing the genetic architecture of CBCs in lactating Holstein cattle. The study provides novel evidence of genetic control of CBC traits in Holstein cattle. The CBC measures had heritability estimates that were generally consistent with those previously identified in beef cattle, with some having estimates larger than 0.50 (RBC and LYMPH), perhaps as a function of breed differences, or the sample size, management, or environmental factors in the current study. Multiple biologically relevant candidate genes were identified that warrant further investigation for causal genes impacting CBC abundance. The GWAS-based QTLs identified overlap with a variety of illness and disease susceptibility traits (e.g., SCS, paratuberculosis, and BRD susceptibility). Significant phenotypic associations have been found between feed intake and CBC counts (results not shown), providing motivation for the determination of the potential utility of CBC traits as proxies for feed intake. Some QTLs identified overlap with QTLs previously identified for production- and efficiency-related traits (e.g., fat, protein and milk yield, ADG, and feed conversion ratio). The results of this study are promising and provide evidence that genomic selection on CBC phenotypes could be feasible.

### 4.1 CBC measures in Holstein cattle are moderately to highly heritable and repeatable

Heritability estimates in the current study for individual CBCs tended to be similar to those previously reported in cattle. Previous reports of the heritability of WBC in beef cattle ranged from 0.31 to 0.47 (Leach et al., 2013; Chinchilla-Vargas et al., 2020), which supports the findings of the current study. Heritability estimates of specific WBC types (i.e., WBC, EOSI, LUC, NEUT, and MONO) were in the range of those reported for both pigs and cattle (Leach et al., 2013; Mpetile et al., 2015; Chinchilla-Vargas et al., 2020), with the exception of BASO and LYMPH, which were considerably larger than those previously reported (0.48 ± 0.05 vs. 0.12—0.23 and 0.68 ± 0.06 vs. 0.15–0.50, respectively). Estimated heritabilities of RBC-related traits also tended to be larger than those previously reported for cattle, while some were similar to those reported in swine (e.g., RBC: 0.56 ± 0.04 vs. 0.62 ± 0.25; Mpetile et al., 2015). Estimates of MCHC, RDW, and MPV heritability were substantially smaller than those previously reported and were near 0 (0.05 ± 0.02, 0.00 ± 0.00, and 0.02 ± 0.01, respectively). An important consideration is that the current study required a transformation of several CBC traits to approximate normality, while previous studies typically did not use transformation measures. It may be possible that these transformations impacted the magnitude of the estimates.

Previous studies have not estimated the repeatability of CBC measures over time. Though samples in this study were taken within a relatively short timeframe (on average 3 weeks apart over a 6-week duration), there was a wide range in repeatability estimates. The mean repeatability of RDW, MPV, and MCHC was low (<0.10), while the repeatability of HGB, WBC, BASO, RBC, and LYMPH was relatively high (>0.50). These findings indicate that some CBC traits with high repeatability may fluctuate little over short time periods, while others with low repeatability may be much more variable. This information about the variability of CBCs over short time periods may be helpful in identifying reasonable time frames to compare CBC measurements in the design of future assays or experiments on dairy cattle. Further research into the point at which a sample is taken for traits that are more variable and the impact that trait variation over time has on trait correlations will be important in determining the informativeness of a candidate indicator trait.

### 4.2 Genome-wide association study and candidate genes for the genetic control of CBCs

#### 4.2.1 White blood cell count

Three genes of interest are located in the window on chromosome 19 that explained the most genetic variance for WBC. Previous research on humans has shown that the *RDM1* gene is involved in mediating DNA damage repair through homologous recombination and the cellular response to a chemotherapy drug (Hamimes et al., 2005; Hamimes et al., 2006; Messaoudi et al., 2007). Moreover, knockdown of this gene reduced the proliferation of tumor cells, increased cell apoptosis, and induced cell cycle arrest (Li et al., 2017; Xu et al., 2018; Chen et al., 2019; Tong et al., 2020). The expression of *RDM1* is correlated with the degree of immune infiltration of immune cells, including macrophages and neutrophils, in a variety of cancer types (Qui et al., 2021). A SNP located within 100 Kb of the *RDM1* gene was associated with interdigital hyperplasia, and another was associated with sole hemorrhage in Holsteins (Sousa Junior et al., 2023). Finally, *RDM1* was found to be significantly downregulated in less feed-efficient beef cattle (Chen et al., 2011). *KANSL1* is an additional gene of interest. A study on human ovarian cancer found that *KANSL1* is amplified and/or rearranged in ovarian cancer, associated with the lymphocyte profile, a biomarker for response to histone deacetylase inhibition, and could potentially drive the expression of genes related to immune response (Fejzo et al., 2020). Lastly, *CDC27* is located in the region identified. This gene has been linked to several diseases including lupus (Shang et al., 2022), pulmonary fibrosis (Qi et al., 2020), and numerous cancers (Ahn et al., 2014; Guo et al., 2015; Xin et al., 2018). Due to the connection of these genes with regulation of immune cells, genes related to immune response, and autoimmune diseases and cancer in humans, it may be worthwhile further investigating their relationship to WBC in cattle.

#### 4.2.2 Red blood cell count

A window located on chromosome 13 explained 6.01% of the genetic variance of RBCs and contained three potential candidate genes, namely, *EYA2*, *OCSTAMP*, and *TP53RK*. These genes have all been tied to blood cancers, including myeloid leukemia and myeloma. One study identified *EYA2* as a potential target for molecular therapy in a subtype of acute myeloid leukemia (Ono et al., 2017), whereas *OCSTAMP* mRNA levels were connected to multiple myeloma (Wang et al., 2020) and *TP53RK* expression is inversely correlated with multiple myeloma survival (Hideshima et al., 2017). Due to acute myeloid leukemia and myeloma being blood cancers, the cancer cells can crowd out healthy blood cells, which can result in decreased RBC and anemia (American Cancer Society, 2018; Mayo Clinic, 2023). Interestingly, *TP53RK* was significantly downregulated in healthy Holstein cattle, compared to those PCR-positive for bovine tuberculosis (Fang et al., 2020), and was expressed at significantly lower levels in foot and mouth disease virus carriers than non-carriers (Zhu et al., 2020).

#### 4.2.3 Hemoglobin abundance and hematocrit percentage

The GWAS regions identified for HGB and HCT were nearly identical, including the window on chromosome 25 that explained the largest portion of genetic variance for both traits. *EPO, AChE*, and *TRF2* genes are located in this window. The protein encoded by the *EPO* gene promotes erythropoiesis in bone marrow. Moreover, the expression of *EPO* is upregulated during hypoxia, which results in increased red blood cell production and an enhanced oxygen-carrying capacity of blood (Schuster et al., 1989; Jelkmann, 1992; Lam et al., 2009). Knock-out of *AChE* in mice resulted in anemia. Additionally, the amount of hemoglobin in the knockout mice was found to be significantly lower. This study also hypothesized that *AChE* may be involved in the regulation of erythroblast-like cell responsiveness to *EPO* (Xu et al., 2019). An additional study on humans by Gupta et al. (2018) found that *AChE* levels were higher in cases of anemia related to the size of the RBCs (i.e., macrocytic and microcytic), suggesting that it may play a role in the maintenance of the shape and integrity of RBCs. This study also reported a negative correlation of *AChE* levels with hemoglobin. The *TFR2* gene is a partner and modulator of the *EPO* receptor gene (*EPOR*) complex and is required for efficient erythropoiesis. Furthermore, knockout of *TFR2* in bone marrow results in higher hemoglobin and red blood cell counts, and researchers speculated that *TFR2* may serve as a control system of RBC number (Nai et al., 2015).

#### 4.2.4 Mean corpuscular volume, mean corpuscular hemoglobin amount, and mean corpuscular hemoglobin concentration

For MCV, MCH, and MCHC, the window that explained the most variance included only one or two genes. Moreover, these genes have not been determined to have functions related to MCV, MCH, MCHC, or related traits. On examining the 1-Mb regions surrounding those identified in the GWAS, some long non-coding RNAs (lncRNAs) for MCV and MCH were observed. Moreover, located just outside of the region identified on chromosome 17 for MCV is the sprout RTK signaling antagonist 1 (*SPRY1*). This gene has been identified as a regulator of red blood cell production during anemia and a transducer of *EPOR* signals (Sathyanarayana et al., 2012). Thus, it may be possible that the lncRNA found may be regulating the nearby gene. For MCHC, the importin 8 (*IPO8*) gene is located roughly 0.1 Mb outside of the identified region. This gene has been associated with increased MCH in mice (Blake et al., 2021) and therefore may be related to MCHC in cattle, but little information is available.

#### 4.2.5 Red cell distribution width

Within the region explaining 1.2% of the genetic variance in RDW are the *ABCG2* and *HERC6* genes. Zhou et al. (2005) reported that the expression of the *ABCG2* gene is upregulated in two murine erythroid cell systems during erythroid differentiation. Moreover, the ABCG2 protein was expressed in mature red blood cells of mice, rhesus monkeys, and humans. Desuzinges-Mandon et al. (2010) reported that the *ABCG2* gene also functions in the cellular export of heme. Similarly, the *HERC6* gene is induced during erythroid differentiation (Maragno et al., 2011). Though these genes have not been related to differences in RDW (i.e., the coefficient of the variation of erythrocyte size), it is possible, given their relationship to erythrocyte differentiation, that they may also influence RDW. Additionally, a mutation in the *ABCG2* gene has previously been identified as having an effect on milk production and composition of Holstein cows (Cohen-Zinder et al., 2005), and HERC6 was found to have increased expression in beef cattle with BRD compared to healthy cattle (Scott et al., 2022).

#### 4.2.6 Platelet count

A gene of interest located in the region explaining the most genetic variance for PLT is *CEP128*. This gene is a risk locus for autoimmune thyroid diseases (Wang et al., 2019). Ijaz et al. (2018) reported that an increase in serum L-thyroxine level, a thyroid hormone, was associated with platelet count. Moreover, thyroid disorders are commonly found in individuals with immune thrombocytopenia (i.e., a deficiency in platelets). The link between *CEP128* and thyroid diseases and the connection between the thyroid and platelets may indicate a potential link between the gene and platelets.

#### 4.2.7 Neutrophil count

The *CXCL12* gene is located in a region explaining 1.3% of the genetic variance of NEUT. This gene is of particular interest as several pieces of evidence exist for its involvement with neutrophils. Metzemaekers et al. (2020) summarizes the roles of chemokines, including *CXCL12*, in relation to neutrophils. Importantly, it is critically involved in neutrophil bone marrow storage and release regulation (Metzemaekers et al., 2020; Cambier et al., 2023). Isles et al. (2019) found that *CXCR4*/*CXCL12* signaling may play a key role in the retention of neutrophils at inflammatory sites. Additionally, *CXCL12* signaling has been shown to enhance neutrophil migration (Cali et al., 2022). Given this clear connection between *CXCL12* and neutrophils, it is a strong candidate gene for neutrophils in cattle.

#### 4.2.8 Lymphocyte count

Two zinc finger and BTB domain-containing genes are located in the window explaining the most genetic variance for LYMPH. This family of genes has been reported to play a key role in B-cell development (Chevrier and Corcoran, 2014). Recently, *ZBTB43* was found to be differentially expressed in human cells after coronavirus infection and was therefore hypothesized to be involved in the cellular response to COVID-19 infection (Mamoor, 2020). Additionally, *ZBTB34* was predicted to be involved in the regulation of immune system processes in brown rats (Vedi et al., 2023). In feedlot cattle, *ZBTB43* was identified as a potential biomarker and candidate disease gene for BRD (Hasankhani et al., 2021), further suggesting it may play a role in the immune system of cattle.

#### 4.2.9 Monocyte count

The *SMOC2* gene is potentially a gene of interest for monocytes in cattle. In a study of humans with heart failure, a negative correlation was found between *SMOC2* and monocytes (Zhou et al., 2023). Moreover, the gene is highly expressed during wound healing (Rocnik et al., 2006). Given that monocytes are required for tissue regeneration and are one of the first responders to tissue injury (Shi and Pamer, 2011; Ogle et al., 2016), it may be possible that *SMOC2* plays a role in the genetic control of monocytes. In beef cross cattle, *SMOC2* was significantly downregulated in healthy cattle compared to those with bovine viral diarrhea virus (Tizioto et al., 2015).

#### 4.2.10 Eosinophil count

The *SULF2* gene is located in the window explaining the most genetic variance for EOSI. This gene is involved in TNF-α signaling and is overexpressed in rheumatoid arthritis (Siegel et al., 2022). Given that rheumatoid arthritis is an autoimmune and inflammatory disease, it is possible that there is a link between *SULF2* and immune-related cells like eosinophils.

#### 4.2.11 Basophil count

Two genes located in the region explaining the most genetic variance for BASO belong to the granzyme family (*GZMA* and *GZMK*). This family of genes is involved in mediating cell death (Chowdhury and Lieberman, 2008), as well as playing a potential role in immune signaling (Cullen et al., 2010). *GZMA* specifically has been shown to have pro-inflammatory activity (Lieberman, 2010). Due to the relationship between BASO and inflammation, a link between granzyme genes and BASO is possible. Aaranday-Cortes et al. (2012) and Bhat et al. (2023) found that *GZMA* was significantly upregulated in cattle with bovine tuberculosis compared to healthy cattle. Interestingly, this gene was more highly expressed in beef cross steers with lower gain than those with higher gain (Lindholm-Perry et al., 2017). A SNP located within 1 Mb of the *GZMK* gene was associated with the Johne’s disease infection status of Holstein cattle (Mallikarjunappa et al., 2018).

#### 4.2.12 Large unstained cells count

Large unstained cells are large peroxidase-negative cells, most often large lymphocytes, virocytes, blasts, and hematopoietic stem cells (Merter et al., 2023). The *TASP1* gene located in the region identified for LUC has been identified as a potential anticancer therapeutic target (Niizuma et al., 2015). Moreover, the gene has been identified as playing a role in filopodia, which is essential during differentiation of innate immune cells and may play a role in the developmental processes of immune cells (Hensel et al., 2022). Soares et al. (2021) reported that the *TASP1* gene was located in a window explaining 0.63% of the genetic variance for subclinical ketosis in first parity Holstein cows.

### 4.3 Study implications and limitations

Despite the limited sample size, this study provides novel information about the heritability and genetic architecture of blood cell traits in dairy cattle. Moreover, sometimes, large populations are not required to obtain strong signals in GWAS analyses of functional traits. Since CBC traits can be impacted by differences in animal health and management styles, these factors should be considered when evaluating CBC traits. The findings are important because they lay the groundwork for future research to evaluate the relationship of CBCs with other traits or identify the underlying causes of variation in these health traits in lactating Holstein cows. In U.S. dairy cattle, the average number of lactations is 2.8, and cows that remain in the herd longer usually have fewer health issues (Shabalina et al., 2019; Hu et al., 2021; Michigan State University Dairy Extension, 2022). Health disorders have a large expense (Bar et al., 2008; Gohary et al., 2016; Liang et al., 2017; Robcis et al., 2023), thus having a significant impact on the economic sustainability of the dairy industry. Moreover, as health directly impacts feed consumption, milk production (Siberski-Cooper et al., 2023), and the efficiency of an animal through nutrient partitioning (Lochmiller and Deerenberg, 2000; Horst et al., 2018; Brown and Bradford, 2021) and increases energy demands (Kvidera et al., 2017), it is worthwhile to examine the genetic relationship of CBCs with longevity, feed intake, and efficiency. Additional studies are needed to evaluate how baseline levels (i.e., normal circulating levels) of CBCs may impact the incidence and severity of illness in dairy cattle.

## 5 Conclusion

Genomic-based heritabilities for CBCs in lactating Holstein dairy cattle were similar to those previously reported in beef cattle. The GWAS results for CBC traits identified many potential candidate genes and overlapped with a host of known GWAS results for disease susceptibility and traits related to animal growth, efficiency, and production. Some of the candidate genes identified may play a role in the immune response (e.g., *RDM1* and *KANSL1*) and the differentiation of RBCs (e.g., *EPO*, *ABCG2*, and *HERC6*). Given the lactation cycle of dairy cattle and the impact it has on immune response, future studies further investigating the relationship of CBC phenotypes with productive life and maintained production efficiency, including assays of cell type functionality, would be beneficial. This study indicates that CBC measures may be useful as proxies for improvement in health, resilience, and feed efficiency.

## Data Availability

The original contributions presented in the study are included in the article/Supplementary Materials; further inquiries can be directed to the corresponding author. Data will also be available in animal QTLdb from April 2024.
