# Supplementary material for: The genetic architecture of complete blood counts in lactating Holstein dairy cows
Source: Front Genet. 2024 Mar 27;15:1360295. doi: 10.3389/fgene.2024.1360295 (PMC11004310; doi:10.3389/fgene.2024.1360295)
Supplement: Supplementary file 1 [file Table1.docx]

**Supplementary Material**

**Supplementary Table 1:** Mean and standard deviation of cell count measures from complete blood counts

| Measure | Mean | Standard deviation |
| --- | --- | --- |
| White blood cells^1^ | 2.07 | 0.24 |
| Neutrophils^1^ | 1.18 | 0.31 |
| Lymphocytes^1^ | 1.33 | 0.32 |
| Monocytes | 0.43 | 0.15 |
| Eosinophils^1^ | -1.67 | 0.71 |
| Basophils^1^ | -2.61 | 0.39 |
| Large unstained cells^1^ | -3.63 | 0.67 |
| Red blood cells | 6.31 | 0.57 |
| Hemoglobin | 10.41 | 0.72 |
| Hematocrit | 22.78 | 1.88 |
| Mean corpuscular volume^1^ | 3.79 | 0.07 |
| Mean corpuscular hemoglobin^1^ | 2.80 | 0.07 |
| Mean corpuscular hemoglobin concentration | 37.47 | 0.71 |
| Red cell distribution width^2^ | 0.50 | 0.00^3^ |
| Platelets | 374.36 | 126.45 |
| Mean platelet volume^2^ | 0.49 | 0.00^3^ |

^1^Log transformed

^2^Box Cox transformed

^3^Small errors at a precision less than two decimal points were estimated but reported as 0.00 due to the precision level of the measurement estimate

**Supplementary Table 2:** Summary of all 1 Mb windows associated with complete blood counts

| Trait | Chromosome | Mb^1^ Window | Number of SNPs in Window | WPPA^2^ | % Genetic Variance Explained |  |
| --- | --- | --- | --- | --- | --- | --- |
| White blood cells | 4 | 23 – 24 | 31 | 0.36 | 1.5 |  |
|  | 9 | 70 – 71 | 25 | 0.24 | 1.1 |  |
|  | 19 | 45 – 46 | 44 | 0.70 | 6.6 |  |
|  | 21 | 61 – 62 | 36 | 0.50 | 3.5 |  |
|  | 29 | 17 – 18 | 23 | 0.39 | 1.3 |  |
| Neutrophils^3^ | 3 | 87 – 88 | 25 | 0.26 | 1.8 |  |
|  | 5 | 38 – 39 | 22 | 0.22 | 1.4 |  |
|  | 27 | 17 – 18 | 37 | 0.31 | 2.8 |  |
|  | 28 | 45 – 46 | 50 | 0.29 | 1.3 |  |
|  | 29 | 8 – 9 | 29 | 0.21 | 1.2 |  |
| Lymphocytes^3^ | 4 | 24 – 25 | 29 | 0.40 | 1.3 | |
|  | 5 | 40 – 41 | 18 | 0.24 | 2.5 | |
|  | 11 | 97 – 98 | 37 | 0.75 | 4.8 | |
|  | 15 | 69 – 70 | 26 | 0.40 | 1.4 | |
|  | 17 | 44 – 45 | 31 | 0.25 | 1.6 | |
|  |  | 46 – 47 | 42 | 0.35 | 1.5 | |
|  | 19 | 45 – 46 | 44 | 0.27 | 1.2 | |
|  | 20 | 2 – 3 | 46 | 0.25 | 1.3 | |
|  | 23 | 28 – 29 | 42 | 0.31 | 1.1 | |
|  |  | 30 – 31 | 31 | 0.30 | 1.1 | |
|  | 29 | 17 – 18 | 23 | 0.53 | 1.7 | |
| Monocytes | 5 | 42 – 43 | 22 | 0.20 | 1.5 | |
|  | 9 | 29 – 30 | 25 | 0.17 | 1.2 | |
|  |  | 102 – 103 | 65 | 0.40 | 3.2 | |
|  | 11 | 92 – 93 | 37 | 0.23 | 1.0 | |
|  | 18 | 5 – 6 | 45 | 0.16 | 1.1 | |
|  | 23 | 13 – 14 | 33 | 0.20 | 1.0 | |
|  | 27 | 41 – 42 | 49 | 0.20 | 1.0 | |
|  | 28 | 45 – 46 | 50 | 0.21 | 1.2 | |

^1^Megabase

^2^Window-based posterior probabilities of association

^3^Log transformed

^4^Box Cox transformed

**Supplementary Table 2 cont.**

| Trait | Chromosome | Mb^1^ Window | Number of SNPs in Window | WPPA^2^ | % Genetic Variance Explained |  |
| --- | --- | --- | --- | --- | --- | --- |
| Eosinophils^3^ | 1 | 34 – 35 | 26 | 0.60 | 3.68 | |
|  | 5 | 28 – 29 | 29 | 0.16 | 1.27 | |
|  | 7 | 78 – 79 | 25 | 0.25 | 1.76 | |
|  | 11 | 14 – 15 | 27 | 0.21 | 1.22 | |
|  | 13 | 76 – 77 | 42 | 0.63 | 5.64 | |
|  | 14 | 6 – 7 | 60 | 0.22 | 1.24 | |
|  | 18 | 27 – 28 | 31 | 0.43 | 2.81 | |
|  | 19 | 4 – 5 | 29 | 0.17 | 1.24 | |
|  |  | 6 – 7 | 36 | 0.23 | 1.78 | |
|  | 28 | 18 – 19 | 44 | 0.27 | 1.48 | |
|  | X | 133 – 134 | 50 | 0.43 | 2.88 | |
| Basophils^3^ | 5 | 40 – 41 | 18 | 0.15 | 1.21 | |
|  | 15 | 42 – 43 | 39 | 0.68 | 3.66 | |
|  | 17 | 44 – 45 | 31 | 0.39 | 3.81 | |
|  | 18 | 43 – 44 | 45 | 0.20 | 1.16 | |
|  |  | 55 – 56 | 39 | 0.45 | 0.29 | |
|  | 19 | 40 – 41 | 44 | 0.39 | 1.58 | |
|  |  | 46 – 47 | 26 | 0.33 | 1.23 | |
|  | 20 | 24 – 25 | 28 | 0.65 | 4.23 | |
| Large Unstained Cells^3^ | 13 | 7 – 8 | 27 | 0.21 | 1.40 | |
| Red Blood Cells | 3 | 58 – 59 | 20 | 0.33 | 1.2 |  |
|  | 11 | 5 – 6 | 38 | 0.32 | 1.2 |  |
|  |  | 13 – 14 | 43 | 0.29 | 1.1 |  |
|  |  | 88 – 89 | 26 | 0.28 | 1.0 |  |
|  | 12 | 86 – 87 | 42 | 0.40 | 1.5 |  |
|  | 13 | 75 – 76 | 44 | 0.94 | 6.0 |  |
|  | 15 | 45 – 46 | 41 | 0.46 | 1.8 |  |
|  | 29 | 33 – 34 | 42 | 0.71 | 5.4 |  |
|  |  | 35 – 36 | 49 | 0.34 | 1.1 |  |

^1^Megabase

^2^Window-based posterior probabilities of association

^3^Log transformed

^4^Box Cox transformed

**Supplementary Table 2 cont.**

| Trait | Chromosome | Mb^1^ Window | Number of SNPs in Window | WPPA^2^ | % Genetic Variance Explained | |
| --- | --- | --- | --- | --- | --- | --- |
| Hemoglobin | 11 | 5 – 6 | 38 | 0.84 | 5.1 |  |
|  | 15 | 4 – 5 | 34 | 0.63 | 2.0 |  |
|  | 25 | 35 – 36 | 45 | 0.61 | 6.0 |  |
|  | X | 136 – 137 | 44 | 0.54 | 2.4 |  |
| Hematocrit | 11 | 5 – 6 | 38 | 0.67 | 3.0 |  |
|  | 15 | 4 – 5 | 34 | 0.50 | 1.3 |  |
|  |  | 77 – 78 | 37 | 0.31 | 1.1 |  |
|  | 25 | 35 – 36 | 45 | 0.82 | 11.9 |  |
|  | X | 136 – 137 | 44 | 0.46 | 2.0 |  |
| Mean Corpuscular Volume^3^ | 5 | 20 – 21 | 26 | 0.58 | 1.3 |  |
|  | 8 | 85 – 86 | 35 | 0.33 | 1.1 |  |
|  | 10 | 0 – 1 | 31 | 0.35 | 1.1 |  |
|  | 17 | 33 – 34 | 36 | 0.37 | 1.2 |  |
| Mean Corpuscular Hemoglobin^3^ | 5 | 20 – 21 | 26 | 0.60 | 1.5 |  |
|  |  | 57 – 58 | 26 | 0.45 | 1.1 |  |
|  | 12 | 14 – 15 | 36 | 0.38 | 1.4 |  |
|  | 19 | 28 – 29 | 55 | 0.52 | 1.1 |  |
|  |  | 50 – 51 | 29 | 0.31 | 1.1 |  |
|  | 22 | 59 – 60 | 63 | 0.50 | 1.4 |  |
| Mean Corpuscular Hemoglobin Concentration | 5 | 79 – 80 | 25 | 0.54 | 5.7 |  |
|  | 12 | 69 – 70 | 41 | 0.26 | 2.3 |  |
|  |  | 73 – 74 | 50 | 0.21 | 1.8 |  |
|  |  | 71 – 72 | 37 | 0.18 | 1.3 |  |
|  |  | 72 – 73 | 14 | 0.15 | 1.2 |  |
|  | 18 | 32 – 33 | 41 | 0.11 | 1.2 |  |
|  | 21 | 19 – 20 | 37 | 0.42 | 3.6 |  |
|  |  | 65 – 65 | 33 | 0.28 | 1.4 |  |

^1^Megabase

^2^Window-based posterior probabilities of association

^3^Log transformed

^4^Box Cox transformed

**Supplementary Table 2 cont.**

| Trait | Chromosome | Mb^1^ Window | Number of SNPs in Window | WPPA^2^ | % Genetic Variance Explained | |
| --- | --- | --- | --- | --- | --- | --- |
| Red Cell Distribution Width^4^ | 2 | 41 – 42 | 21 | 0.35 | 1.4 |  |
|  | 6 | 36 – 37 | 73 | 0.24 | 1.2 |  |
|  |  | 37 – 38 | 58 | 0.54 | 4.4 |  |
|  | 14 | 19 – 20 | 34 | 0.25 | 1.5 |  |
|  | 16 | 22 – 23 | 25 | 0.36 | 4.4 |  |
|  | X | 137 – 138 | 44 | 0.43 | 2.6 |  |
| Platelets | 10 | 92 – 93 | 39 | 0.28 | 3.2 |  |
|  | 27 | 35 – 36 | 42 | 0.40 | 3.1 |  |
|  | 28 | 15 – 16 | 36 | 0.23 | 1.0 |  |
| Mean Platelet Volume^3^ | 5 | 12 – 13 | 57 | 0.63 | 3.1 | |
|  |  | 118 – 119 | 62 | 0.31 | 1.5 | |
|  |  | 119 – 120 | 49 | 0.47 | 1.5 | |
|  | 8 | 75 – 76 | 30 | 0.33 | 1.9 | |
|  | 9 | 77 – 78 | 32 | 0.61 | 4.6 | |
|  | 11 | 24 – 25 | 40 | 0.40 | 1.3 | |
|  | 13 | 70 – 71 | 36 | 0.25 | 1.0 | |
|  | 22 | 36 – 37 | 30 | 0.34 | 1.6 | |
|  | 24 | 9 – 10 | 31 | 0.45 | 3.1 | |
|  | 28 | 19 – 20 | 28 | 0.47 | 2.4 | |
|  | X | 9 – 10 | 27 | 0.39 | 1.7 | |
|  |  | 10 – 11 | 30 | 0.24 | 1.2 | |

^1^Megabase

^2^Window-based posterior probabilities of association

^3^Log transformed

^4^Box Cox transformed

**Supplementary Table 3:** Quantitative trait loci (**QTL**) associated with illness, disease resilience, and production and efficiency traits that overlap the QTL explaining the most genetic variance for each complete blood count (**CBC**) measure found in the present study

| Measure | Chromosome | Megabase | QTL Span (Mbp) | Peak SNP | Trait |
| --- | --- | --- | --- | --- | --- |
| White blood cells^1^ | 19 | 44 | 44.5 – 44.5  44.5 – 44.5  49.9 – 50.0 | rs41916457  rs109158773 rs41915550  rs41916457 | Milk fat percentage (daughter deviation)  Milk conjugated linoleic acid content |
|  |  | 45^3^ | 49.9 – 50.0  45.9 – 45.9 | rs41636123  rs41577559  rs136009289  rs133533654 | Milk conjugated linoleic acid content  Milk riboflavin content |
| Neutrophils^1^ | 27 | 16 | 16.2 – 16.2  16.3 – 16.3  16.6 – 16.6  16.7 – 16.7  16.6 – 16.6  16.6 – 16.6  16.6 – 16.6  16.6 – 16.6  16.6 – 16.6  16.6 – 16.6 | rs42851890  rs135829876  rs42111521  rs42112443  rs135718129  rs43083444  rs42110122  rs41649184  rs41649184  rs41649184  rs41649184  rs41649184  rs41649184 | Milk fat percentage (daughter deviation)  Milk potassium content  Milk fat percentage  Milk fat yield  Milk protein percentage  Milk protein yield  Length of productive life  Net merit |
|  |  | 17^3^ | 17.1 – 17.1  17.2 – 17.2 | rs109154295  rs41645652 | Average daily gain  Body weight gain |
|  |  | 18 | 18.4 – 18.4  18.6 – 18.6  18.7 – 18.7 | rs41567833  rs43059887  rs42843197 | Average daily gain  Dry matter intake  Milk kappa-casein percentage |

^1^Log transformed; ^2^Box Cox transformed; ^3^1 Mb window explaining the most genetic variance for the CBC measure

**Supplementary Table 3 cont.**

| Measure | Chromosome | Megabase | QTL Span (Mbp) | Peak SNP | Trait |
| --- | --- | --- | --- | --- | --- |
| Monocytes | 9 | 101 | 101.7 – 101.7 | rs109383352 | Milk alpha-S2-casein percentage |
|  |  | 102^3^ | 102.1 – 102.2  102.2 – 102.2  102.2 – 102.2  102.5 – 102.5 | rs109603023  rs134789789  rs134754817  rs137835498  rs137418234  rs132902810  rs134581769  rs134754817  rs137835498  rs132902810  rs134581769  rs41617598 | Bovine respiratory disease susceptibility  Milk alpha-S2-casein percentage  Milk phosphorylated alpha-S2-casein percentage  Length of productive life |
|  |  | 103 | 103.4 – 103.4  103.8 – 103.8 | rs41604520  rs109338526 | Length of productive life  Body weight gain |
| Eosinophils^1^ | 13 | 75 | 75.9 – 75.9 | rs42056588 | Milk glycosylated kappa-casein percentage |
|  |  | 76^3^ | 76.0 – 76.0  76.0 – 76.0  76.4 – 76.4 | rs108951661  rs41587252  rs108951661  rs41587252  rs109235034 | Length of productive life  Net merit  Body weight gain |
|  |  | 77 | 77.4 – 77.4  77.6 – 77.6  77.9 – 77.9  77.9 – 77.9 | rs41634068  rs137320993  rs109123247  rs41710487  rs109934030  rs109934030 | Somatic cell score  Clinical mastitis |

^1^Log transformed; ^2^Box Cox transformed; ^3^1 Mb window explaining the most genetic variance for the CBC measure

**Supplementary Table 3 cont.**

| Measure | Chromosome | Megabase | QTL Span (Mbp) | Peak SNP | Trait |
| --- | --- | --- | --- | --- | --- |
| Basophils^1^ | 20 | 23 | 23.0 – 23.0  23.1 – 23.1  23.2 – 23.2 | rs29013716  rs29026076  rs137351689 | Clinical mastitis  Milk yield  Length of productive life |
|  |  | 24^3^ | 24.2 – 24.2  24.7 – 24.7  24.2 – 24.2  24.5 – 24.5  24.8 – 24.8  24.9 – 24.9  24.4 – 24.4  24.6 – 24.6 | rs109440459  rs41638409  rs132994971  rs110372501  rs110147986  rs109348299  rs109832360  rs110426517  rs110631962  rs109284633  rs133373112  rs134898410  rs29023196  rs136142926  rs109175103  rs110956125  rs109107106  rs135779439  rs110550258  rs132641629  rs135486682  rs41596520  rs29022923 | Milk fat yield (daughter deviation)  Length of productive life  Milk fat percentage (daughter deviation)  Milk fat yield |

^1^Log transformed; ^2^Box Cox transformed; ^3^1 Mb window explaining the most genetic variance for the CBC measure

**Supplementary Table 3 cont.**

| Measure | Chromosome | Megabase | QTL Span (Mbp) | Peak SNP | Trait |
| --- | --- | --- | --- | --- | --- |
| Basophils^1^  continued | 20 | 25 | 25.0 – 25.0  25.5 – 25.5  25.7 – 25.7  25.6 – 25.6 | rs109397111  rs110685186  rs136450650  rs134138031  rs137594841  rs133549221  rs109854193  rs111010393  rs109247499 | Length of productive life  Somatic cell score |
| Large unstained cells^1^ | 13 | 6 | 6.3 – 6.3  6.3 – 6.3  6.5 – 6.5  6.3 – 6.3 | rs41659130  rs41659130  rs41660031  rs29017378  rs41659130 | Somatic cell score  Length of productive life  Net merit |
|  |  | 7^3^ | 7.0 – 7.0  7.0 – 7.0 | rs29023285  rs29023285 | Length of productive life  Net merit |
|  |  | 8 | 8.8 – 8.8 | rs41678980  rs135856735 | Milk iron content |
| Red blood cells | 13 | 74 | 74.0 – 74.0  74.9 – 74.9  74.7 – 74.7  74.7 – 74.7  74.7 – 74.7  74.7 – 74.7  74.7 – 74.7  74.7 – 74.7  74.8 – 74.8  74.7 – 74.7  74.8 – 74.8 | rs134142955  rs41633492  rs41710700  rs109747489  rs109747489  rs109747489  rs109747489  rs109747489  rs109747489  rs41711496  rs109747489  rs41711496 | Milk kappa-casein percentage  Milk fat percentage  Milk fat yield  Milk protein percentage  Milk protein yield  Milk yield  Length of productive life  Net merit |

^1^Log transformed; ^2^Box Cox transformed; ^3^1 Mb window explaining the most genetic variance for the CBC measure

**Supplementary Table 3 cont.**

| Measure | Chromosome | Megabase | QTL Span (Mbp) | Peak SNP | Trait |
| --- | --- | --- | --- | --- | --- |
| Red blood cells  continued | 13 | 74 | 74.2 – 74.2  74.2 – 74.2 | rs43024409  rs43024409 | Metabolic body weight  Average daily gain |
|  |  | 75^3^ | 75.0 – 75.0  75.0 – 75.0  75.0 – 75.0  75.0 – 75.0  75.0 – 75.0  75.0 – 75.0  75.9 – 75.9 | rs110364197  rs110364197  rs110364197  rs110364197  rs110364197  rs110364197  rs42056588 | Milk fat percentage  Milk fat yield  Milk protein percentage  Milk protein yield  Milk yield  Net merit  Milk glycosylated kappa-casein percentage |
|  |  | 76 | 76.0 – 76.0  76.0 -76.0  76.0 – 76.0  76.4 – 76.4 | rs41587252  rs108951661  rs41587252  rs108951661  rs41587252  rs109235034 | Somatic cell score  Length of productive life  Net merit  Body weight gain |
| Hemoglobin and Hematocrit | 25 | 34 | 34.1 – 34.1  34.3 – 34.3  34.5 – 34.5  34.6 – 34.6  34.8 – 34.8  34.8 – 34.8 | rs110288675  rs109194151  rs109405213  rs111005382  rs110266411  rs41567529  rs41567529 | Milk calcium content  Milk potassium content Milk fat yield (daughter deviation)  Milk fat yield  Milk trans-fatty acid content |
|  |  | 35^3^ | 35.0 – 35.0 | rs134690880  rs135804441  rs137793184  rs110096656  rs133311984  rs132649394  rs135509245 | Milk linoleic acid content |

^1^Log transformed; ^2^Box Cox transformed; ^3^1 Mb window explaining the most genetic variance for the CBC measure

**Supplementary Table 3 cont.**

| Measure | Chromosome | Megabase | QTL Span (Mbp) | Peak SNP | Trait |
| --- | --- | --- | --- | --- | --- |
| Hemoglobin and Hematocrit continued | 25 | 35^3^ | 35.0 – 35.0  35.1 – 35.1  35.5 – 35.5  35.6 – 35.6  35.7 – 35.7  35.7 – 35.7 | rs134690880  rs110096656  rs132649394  rs137793184  rs109468931  rs208606059  rs109480808  rs43726576  rs110452329  rs109410006 | Milk potassium content  Average daily gain  Milk fat yield  Milk fat yield (daughter deviation)  Milk fat percentage (daughter deviation) |
| Mean corpuscular volume^1^ and  Mean corpuscular hemoglobin^1^ | 5 | 20^3^ | 20.8 – 20.8 | rs108948843 | Milk fat yield (daughter deviation) |
|  |  | 21 | 21.0 – 21.0  21.1 – 21.1  21.7 | rs135863760  rs109952851  rs133198838  rs377953581  rs110483973 | Milk potassium content  Milk lactose content  Milk myristoleic acid content |
| Red cell distribution width^2^ | 16 | 21 | 21.1 – 21.1  21.6 – 21.6  21.7 – 21.7  21.1 – 21.1  21.6 – 21.6  21.7 – 21.7  21.1 – 21.1  21.6 – 21.6  21.7 – 21.7  21.6 – 21.6  21.6 – 21.6 | rs110034639  rs41635427  rs110838190  rs110034639  rs41635427  rs110838190  rs110034639  rs41635427  rs110838190  rs41635427  rs41635427 | Somatic cell score  Length of productive life  Net merit  Average daily gain  Body weight gain |

^1^Log transformed; ^2^Box Cox transformed; ^3^1 Mb window explaining the most genetic variance for the CBC measure

**Supplementary Table 3 cont.**

| Measure | Chromosome | Megabase | QTL Span (Mbp) | Peak SNP | Trait |
| --- | --- | --- | --- | --- | --- |
| Red cell distribution width^2^ continued | 16 | 22^3^ | 22.0 – 22.0  22.1 – 22.1  22.2 – 22.2  22.3 – 22.3  22.3 – 22.3  22.3 – 22.3 | rs133566900  rs136125404  rs137698507  rs132802568  rs135686213  rs132980165  rs110532366  rs110532366  rs110532366 | Milk iron content  Somatic cell score  Length of productive life  Net merit |
|  |  | 23 | 23.6 – 23.6  23.7 – 23.7 | rs110532366  rs137245725 | Feed conversion ratio |
| Mean corpuscular hemoglobin concentration | 5 | 78 | 78.5  78.6 – 78.6 | rs109789610  rs109527842  rs110121055  rs135967189  rs109207842  rs110176858  rs135461267  rs109946089  rs109946089  rs110285991  rs136674883  rs137439889  rs109484782  rs109840973 | Bovine tuberculosis susceptibility |
|  |  | 80 | 81.0 – 81.0 | rs29023629 | M. paratuberculosis susceptibility |
| Platelets | 10 | 91 | 91.2 – 91.2 | rs42931883 | Body weight gain |
|  |  | 92^3^ | 92.3 – 92.3 | rs43648326 | Body weight gain |

^1^Log transformed; ^2^Box Cox transformed; ^3^1 Mb window explaining the most genetic variance for the CBC measure

**Supplementary Table 3 cont.**

| Measure | Chromosome | Megabase | QTL Span (Mbp) | Peak SNP | Trait |
| --- | --- | --- | --- | --- | --- |
| Mean platelet volume^2^ | 9 | 76 | 76.0 – 76.0  76.1 – 76.1  76.2 – 76.2  76.1 – 76.1  76.2 – 76.2  76.1 – 76.1  76.2 – 76.2  76.1 – 76.1  76.2 – 76.2  76.1 – 76.1  76.2 – 76.2  76.1 – 76.1  76.2 – 76.2  76.4 – 76.4 | rs41594180  rs41663519  rs109222277  rs41663519  rs109222277  rs41663519  rs109222277  rs41663519  rs109222277  rs41663519  rs109222277  rs41663519  rs109222277  rs136031255  rs133031259  rs134503747 | Average daily feed intake  Milk fat percentage  Milk fat yield  Milk protein percentage  Milk protein yield  Length of productive life  Net merit  Milk beta-lactoglobulin percentage |
|  |  | 77^3^ | 77.3 – 77.3  77.5 – 77.5  77.8 – 77.8 | rs134113332  rs137652129  rs132907269  rs110475796  rs41666493 | Milk beta-lactoglobulin percentage  Lactation persistency  Milk protein yield (daughter deviation) |
|  |  | 78 | 78.7 – 78.7  78.8 – 78.8 | rs133962107  rs134511641  rs134315345 | Metabolic body weight  Milk beta-lactoglobulin percentage |

^1^Log transformed; ^2^Box Cox transformed; ^3^1 Mb window explaining the most genetic variance for the CBC measure
